# Knowledge, Attitudes, and Practices Regarding Influenza and Pertussis Immunization During Pregnancy in Greece †

**DOI:** 10.3390/vaccines13040347

**Published:** 2025-03-25

**Authors:** Panagiota Georgia Maltezou, Eleni Kourkouni, Dimitra Kousi, Christos Hadjichristodoulou, Aikaterini Dadouli, Despoina Briana, Vassiliki Papaevangelou

**Affiliations:** 1 Third Department of Pediatrics, University Hospital Attikon, 124 62 Athens, Greecevpapaev@gmail.com (V.P.); 2 Collaborative Center for Clinical Epidemiology and Outcomes Research (CLEO), 154 51 Athens, Greece; 3 Laboratory of Hygiene and Epidemiology, University of Thessaly, 382 21 Larissa, Greece

**Keywords:** maternal immunization, influenza, pertussis, whooping cough, maternal vaccine uptake, cocooning

## Abstract

Background/Objectives: Vaccination against influenza and pertussis in pregnant women protects the mother and child through the transfer of protective antibodies across the placenta. However, pregnant women’s vaccine hesitancy is a major barrier to achieve satisfactory vaccination coverage in many developed countries. Methods: Greek pregnant women’s vaccination knowledge, attitudes, and practices were recorded. Structured questionnaires were administered to mothers of infants under the age of 12 months through their pediatricians. Sampling across the country’s districts was applied to achieve geographic representativeness. Results: Questionnaires from 474 mothers were collected. Their mean age was 34 (±5) years. Vaccination uptake was 16.8% and 45.7%, for pertussis and influenza, respectively. During their recent pregnancy, 68.9% and 27.1% of the responders had been informed by their gynecologists regarding influenza and pertussis maternal immunization, respectively, indicating that gynecologists miss out on informing a significant rate of pregnant women. According to multiple logistic regression, women who gave birth during spring (OR: 2.29 vs. winter delivery, *p* = 0.042) and those with an MSc or PhD (OR: 2.93 vs. school graduates, *p* = 0.015) were more likely to receive influenza vaccination. Factors favoring influenza vaccination included doctor’s recommendation (OR: 18.86, *p* < 0.001), being not/somewhat afraid of potential vaccine side effects during pregnancy (OR: 2.09, *p* = 0.012), considering the flu as relatively/very dangerous during pregnancy (OR: 8.05, *p* < 0.001), and considering the flu vaccine as relatively/completely safe (OR: 4.37, *p* < 0.001). Doctor’s recommendation (OR: 29.55, *p* < 0.001) and considering pertussis a relatively/very serious risk to the mother’s health during pregnancy (OR: 6.00, *p* = 0.002) were factors associated with pertussis vaccination during pregnancy. Conclusions: The education of both expectant mothers and obstetricians is urgently needed in order to increase immunization coverage during pregnancy. The low influenza vaccination coverage among women delivering during winter and low pertussis immunization rates, in combination with low recommendation rates for both vaccines, strongly indicate that Greek obstetricians focus on maternal health alone. Their perspectives play an instrumental role in vaccine acceptance during pregnancy, shaping the immunization inclusion maps.

## 1. Introduction

Vaccination has successfully decreased the mortality and morbidity of several vaccine-preventable infectious diseases [1]. However, along with the significant public health benefit achievement, safety concerns have arisen, primarily due to the extensive use of media and other sources of information [2]. The World Health Organization (WHO) defines vaccine hesitancy as an overall behavior influenced by factors related to (1) issues of trust, (2) questions of controversy (the value of the vaccine and the necessity of vaccination), and (3) access issues regarding vaccine delivery and vaccination systems [3,4].

Pregnant women’s vaccination is an important tool for protecting fetal and neonatal health [5]. During pregnancy, maternal antibodies are transmitted to the fetus via the placenta, while secretory IgA antibodies are transferred to the infant through breastfeeding. The fetus, and, later, the newborn, is thus protected from life-threatening diseases during the first months of life, when both cellular and humoral immunity are weak, and infant immunization has not begun [6]. Notably, influenza vaccination is important for maternal health as well [7].

Pregnancy-related influenza vaccination recommendations have been in place since the 1960s, while the Centers for Disease Control and Prevention (CDC) advised for vaccination during pregnancy against tetanus, diphtheria, and acellular pertussis (Tdap) in 2011 [8,9]. Both vaccines are regarded as safe during pregnancy [10]. In the United States, coverage estimates for both vaccines are still below ideal levels despite CDC recommendations [11,12]. A recent overview of European policies on maternal immunization documents that several European nations, in accordance with WHO guidelines, have customized vaccination programs for expectant mothers [13]. However, the strategies used in different European countries and, consequently, the implementation efficacy vary [13].

According to a recent international survey on vaccine refusal, Greece was ranked among the top 10 nations with the lowest rates of positive sentiment [14,15,16]. A study conducted in Western Greece in 2017 documented high awareness regarding influenza and pertussis among pregnant women. However, respondents lacked knowledge as far as the respective vaccines, and their safety were concerned [17]. Vaccination coverage rates among pregnant women in Greece have not been studied nationwide and remain grossly unknown since there is no national vaccination registry.

The aim of this study is to estimate vaccination coverage among Greek pregnant women in a stratified sample. Additionally, this study aspired to ascertain the potential causes of inadequate vaccination and identify knowledge and awareness gaps among pregnant women.

## 2. Materials and Methods

A cross-sectional national study assessing the knowledge, attitudes, and practices [Knowledge, Attitude and Practices (KAP) study] of pregnant women was conducted between December 2020 and May 2022. Structured questionnaires were distributed to mothers of infants <12 months residing in Greece. The initial version of the questionnaire was based on respective studies conducted in Europe during the last years. The translation-back translation method was applied, and the tool was evaluated by pediatric infectious diseases and epidemiology experts. A pilot study was conducted by distributing the questionnaires to ten participants to assess the validity and internal consistency of the tool. Cronbach’s alpha (denoted as α) was used as the statistical measure to assess the internal consistency or reliability of the questionnaire. Following pilot study data processing, Cronbach’s alpha was calculated at 0.5. A question was removed, and the Cronbach’s alpha was calculated at 0.645, providing satisfactory instrument reliability [18]. Sampling, considering the geographically distributed administrative districts of the country as layers and pediatricians as clusters, was carried out. For each pediatrician who was randomly selected, ten (10) mothers were asked to participate during the opening hours of their medical offices. All mothers of infants younger than 12 months of age were considered eligible for the study. Duration of pregnancy, number of offsprings, parity, ethnicity, education level, or month of delivery were not factors that determine exclusion criteria for our subjects. The questionnaires were completed independently by the mothers.

### 2.1. Statistical Analysis

The statistical analysis of the collected data was performed by the Center for Clinical Epidemiology and Outcomes Research (CLEO) in Athens. The sample size required was calculated considering the number of annual live births according to national registries (N = 86,550), the confidence level at 95%, the margin of error at 5% and the estimated maternal vaccination rate for influenza at 50%. Therefore, answers should be collected from 383 mothers who had recently given birth. Estimating that the response rate would be as high as 65%, questionnaires should be distributed to 590 mothers [19].

Categorical data are presented with absolute and relative (%) frequencies, while quantitative data are presented with mean, standard deviation (SD), median, interquartile range (IQR), minimum, and maximum value. Normality of quantitative data was evaluated graphically with histograms. The X 2 test of independence, Fisher’s exact test (where applicable), the independent-samples t-test, and the non-parametric Mann–Whitney test were used to identify potential factors related to vaccination coverage of pregnant women against influenza and whooping cough. Multiple logistic regression was performed to assess the effect of these factors on pregnant women’s vaccination coverage. The odds ratio (OR) along with the 95% confidence intervals (CI) were used to present the results. The statistical significance level was set at 5%. STATA SE v18 statistical software was used for the analysis.

### 2.2. Evaluation of Knowledge

Each mother’s knowledge score was calculated based on responses given in 9 questions (min–max: 0–9). A score was determined only for the participants that had responded to all nine questions (Q3–Q11). The Supplementary Materials contains a description of the scoring procedure for each question. When the mother’s knowledge score fell between 0 and 3, it was considered low; between 4–6, it was considered moderate; and, between 7–9, it was considered high.

## 3. Results

**Figure 1:**
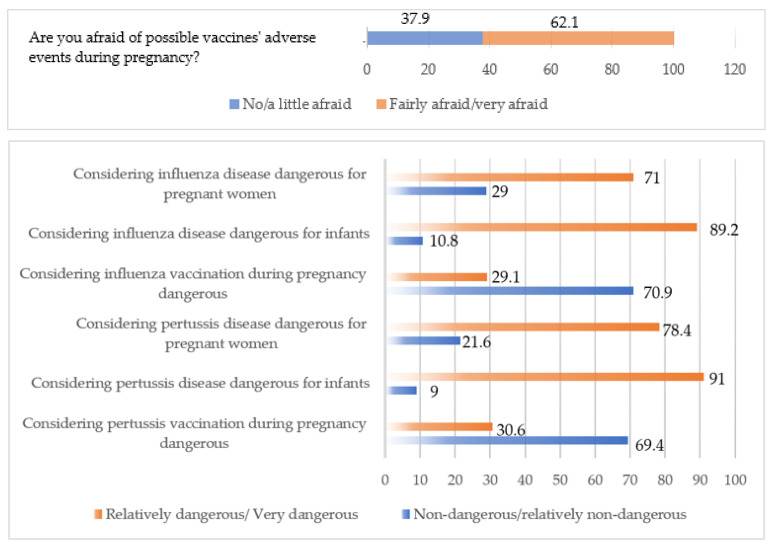
Descriptive analysis depicted as % rates of the maternal attitudes regarding influenza and pertussis vaccinations.

**Figure 2:**
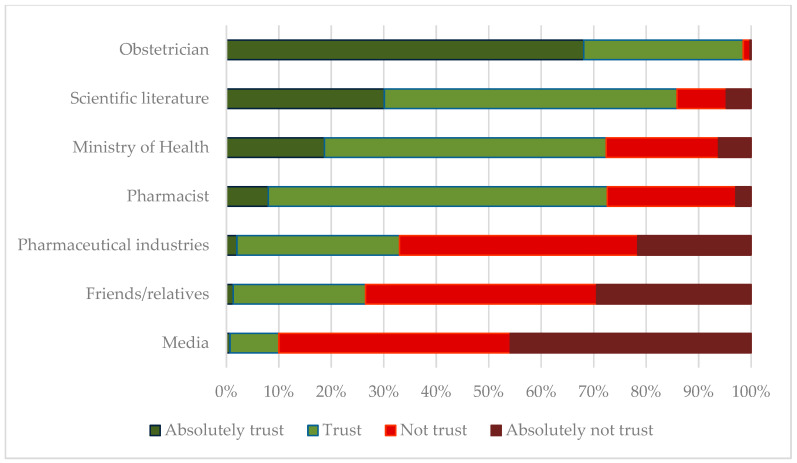
Level of trust reported by new mothers in different sources of information in vaccine-related issues.

Initially, 6 pediatricians working in 6 different public hospitals and 50 pediatricians across all geographic regions working in private medical offices were recruited. Six hundred questionnaires were distributed. The response rate was higher than expected, with 474 mothers participating in the survey (response rate = 79%). Table 1 depicts the demographics of the mothers who responded to the questionnaire (N = 474). Figure 1 describes maternal attitudes regarding influenza and pertussis vaccinations while Figure 2 depicts the level of trust among different sources of information on vaccine-related issues.

### 3.1. Influenza Maternal Vaccination

The reported rate of influenza vaccination during their index pregnancy was 45.7%. Most women declared that their gynecologist had informed them about the need to be vaccinated against influenza during their pregnancy (68.9%), and perceived that influenza is associated with a risk to infants and themselves (89.2% and 71.1%, respectively). However, 29.2% considered influenza vaccination during pregnancy relatively/very dangerous. Out of 250 mothers who had an older child, only 70 (28%) had been vaccinated against influenza while being pregnant with their older child.

Non-vaccination was mainly attributed to a lack of doctor’s recommendation (62.5%) and to the perception that pregnancy is not a risk factor for severe disease (34.2%). Nevertheless, 39.6% of mothers stated that it is likely they would get vaccinated in a following pregnancy, while 25.7% stated that they would do so only after their Healthcare Practitioner’s (HCP’s) recommendation.

Factors associated with influenza vaccine uptake during pregnancy (crude logistic regression) were maternal age, nationality, education level, and occupation, as well as insurance type, season of labor, family status, and the HCP’s recommendation. The vaccination rates had also been impacted by maternal knowledge about influenza and mothers’ beliefs regarding the risks associated with diseases and vaccinations for expectant mothers and fetuses (Table 2).

Following multiple logistic regression, expectant mothers were more likely to be vaccinated if (a) their HCP’s recommended vaccination (OR: 18.86, 95% CI: 8.61–41.31, *p* < 0.001); (b) they were expected to give birth during the spring compared to winter (OR: 2.29, 95% CI: 1.03–5.07, *p* = 0.042); (c) they were holding a PhD/MSc (OR: 2.93, 95% CI: 1.23–7.00, *p* = 0.015); (d) they considered influenza to be relatively dangerous/very dangerous for the mother’s health (OR: 8.05, 95% CI: 3.81–17.03, *p* = 0.012); (e) they considered the influenza vaccine to be non-dangerous/relatively non-dangerous during pregnancy (OR: 4.37, 95% CI: 2.27–8.41, *p* < 0.001); and they were not/a little afraid of possible vaccine side effects during pregnancy (OR: 2.09, 95% CI: 1.18–3.70, *p* = 0.012).

### 3.2. Pertussis Maternal Vaccination

The maternal pertussis vaccination rate was 16.8% (78/474). Most women (72.9%) reported that they did not receive any relevant information from their HCPs. Many women perceived pertussis to be relatively dangerous/very dangerous for pregnant women and for the infant (78.3% and 91%, respectively). However, maternal pertussis vaccination was considered relatively/very dangerous by 143 responders (30.6%). Among the multiparous mothers (N = 250), the vast majority (233/247; 94.3%) reported an absence of vaccination in their previous pregnancies. Table 3 depicts the results of the crude logistic regression, according to which factors that were significantly associated with maternal pertussis vaccine uptake were parity, doctor’s recommendation, fear of vaccines’ adverse events, and considering whooping cough relatively dangerous/very dangerous for the pregnant woman.

## 4. Discussion

This is the first study to document maternal knowledge, attitudes, and practices regarding influenza and pertussis vaccinations in Greece, in which a large sample was stratified among different geographical regions. It was conducted following the COVID-19 pandemic. Gkentzi et al. have also recorded the vaccine uptake during pregnancy in western Greece and have ended up with similar conclusions, highlighting the urgent need for education and awareness [17]. Results from the present cross-sectional study show that pregnant women’s vaccination rates for pertussis and influenza are far below ideal. Primary causes identified include the absence of HCP’s recommendations and low maternal knowledge regarding both the risk of these infections for themselves and their offspring, and the effectiveness and safety of the relevant vaccination. Importantly, the overall low pertussis vaccine uptake and the low influenza vaccination rates among women who gave birth in winter show that, in Greece, obstetricians focus on maternal health and tend to overlook the importance of protecting the offspring as well.

Therefore, it is imperative that we increase the awareness on maternal vaccination among both obstetricians and women of childbearing age. Midwives should be addressed as well, since they provide maternity care and have a significant impact on women’s decisions regarding vaccinations [19,20]. A recording from Greece conducted by Taskou et al. highlights the crucial role HCPs have in the antenatal influenza immunization. Their role was even more significant during the SARS-CoV-2 pandemic, when the protection of public health and preventive measures’ implementation were of major importance [21]. The main challenge when addressing the low vaccination acceptance among pregnant women is vaccine hesitancy, mainly attributed to the fear of possible vaccines’ adverse events for pregnant woman and the fetus. Therefore, there is an urgent need to empower both obstetricians and midwives through education, aiming to increase their confidence in advocating for the efficacy and safety of maternal immunization and addressing women’s doubts and fears [19,22,23]. Pediatricians play a fundamental role in promoting maternal vaccination, given their primary responsibility for the child’s health and their position as trusted healthcare providers for families and new mothers. Their influence extends beyond pediatric care, as they are key advocates for the safety and efficacy of maternal immunization, thereby facilitating informed decision-making that supports the well-being of both mother and child [24].

As per influenza maternal vaccination, approximately half of the respondents reported that they received the vaccine during their recent pregnancy. Women who received a recommendation from their HCP were 18.86 times more likely to get vaccinated. A prior systematic review and meta-analysis that was published in 2020 concluded that pregnant women who received a recommendation from a healthcare professional were 10–12 times more likely to receive a pertussis or influenza vaccination. When individuals believed there was a possibility of vaccine-induced side effects, their likelihood of getting vaccinated was five times lower [24]. An Australian study found that women were 20 times more likely to get the influenza vaccine if a healthcare worker had recommended it to them [25,26]. Despite this, most HCPs worldwide continue to doubt maternal immunization, discouraging pregnant women from getting vaccinated [27]. These recordings and literature reviews emphasize how crucial it is to give medical professionals the knowledge and autonomy to clearly advise women and families [28,29].

Vaccination against pertussis in each pregnancy is recommended in most developed countries, given the fact that whooping cough has a significant rate of mortality and morbidity during infancy and maternal vaccination is the only tool to protect the most vulnerable young infants [30,31]. Importantly, recently, there has been a significant increase in pertussis cases among infants in Europe, with higher morbidity and mortality rates to be documented in young infants [32,33]. In a recent systematic review, premature delivery, low maternal age, and a lack of public insurance were identified as the key factors linked to low maternal pertussis vaccination acceptance [15]. Notably, HCPs’ advice was important, since mothers who had received the relevant information were significantly more likely to get vaccinated [15].

The main limitations of this KAP study concern participation bias. It is possible that respondents are more likely to be mothers with a positive attitude towards vaccination. Similarly, the socioeconomic status of the responders constitutes another limitation. Despite efforts, illiterate women were underrepresented in this study. Unfortunately, the interviewers were not able to document demographic data from non-responders that would be helpful in depicting this kind of bias in our recording. Finally, vaccine uptake was not studied in association with pediatrician’s point of view. Pediatricians are healthcare providers who are familiar with vaccinations and may influence families’ practices, especially in cases of multiparous mothers.

Influenza and pertussis vaccine uptake during pregnancy remains low among Greek expectant mothers, mostly attributed to lack of HCP’s recommendations and suboptimal maternal awareness regarding the risks of these two diseases for maternal, fetal, and neonatal health. Additionally, the efficacy and safety of the respective available vaccines are doubted by many pregnant women. Obstetricians are the main healthcare providers responsible for maternal health and, thus, their attitudes play a significant role in maternal immunization. Pediatricians’ role places them in a unique position as well, advocating for the proven safety and effectiveness of maternal immunization, ultimately guiding parents toward informed choices that promote the health of both the mother and child. Their education regarding vaccination during pregnancy needs to be consistent and targeted to increase vaccination rates among pregnant women and support the proper application of preventive medicine.

## 5. Conclusions

In conclusion, maternal immunization is a critical component of public health that protects both the mothers and infants from preventable diseases. Despite its proven benefits, significant gaps remain in the education of both women of reproductive age and in the engagement of healthcare practitioners regarding its importance. Addressing these gaps is essential for improving immunization rates and overall maternal and neonatal health outcomes.

## Figures and Tables

**Table 1:** Maternal characteristics.

Characteristic	N (%)
**Maternal age (groups)** (19 missing)	
<25	21 (4.6%)
25–29	73 (16.0%)
30–34	143 (31.5%)
≥35	218 (47.9%)
**Nationality ** (5 missing)	
Greek	395 (84.2%)
Other	74 (15.8%)
**Number of children** (58 missing)	
1	184 (44.3%)
2	183 (44.0%)
3	40 (9.6%)
4	8 (1.9%)
5	1 (0.2%)
**Season of labor ** (30 missing)	
Winter	103 (23.2%)
Spring	113 (25.4%)
Summer	114 (25.6%)
Autumn	115 (25.8%)
**Living region** (21 missing)	
Athens	169 (37.2%)
Another Greek city	177 (39.2%)
Another Greek town	107 (23.6%)
**Family state ** (4 missing)	
Unmarried	28 (6.0%)
Married/cohabitation agreement	435 (92.5%)
Divorced/estranged	7 (1.5%)
**Insurance** (6 missing)	
No	17 (3.6%)
Yes	451 (96.4%)
**Are you considered a high-risk group?** (50 missing)	
No	414 (97.6%)
Yes	10 (2.4%)
**Maternal education level** (78 missing)	
School graduate	146 (36.7%)
Technical school graduate	81 (20.6%)
University graduate	93 (23.5%)
MSc/PhD ^§^	76 (19.2%)
**Mother’s profession** (1 missing)	
Public worker	73 (15.4%)
Private worker	203 (42.9%)
Freelancer	70 (14.8%)
Unemployed	56 (11.8%)
Other	71 (15.1%)
**Healthcare practitioner’s (HCP’s) recommendation regarding influenza vaccination during pregnancy** (4 missing)	
No	146 (31.1%)
Yes	324 (68.9%)
**HCP’s recommendation regarding pertussis vaccination during pregnancy** (6 missing)	
No	341 (72.9%)
Yes	127 (27.1%)
**Knowledge score ** (35 missing)	N = 439
Mean [Standard Deviation (SD)]	7 (2)
Median [Interquartile Range (IQR)]	8 (7–9)
Min–Max	0–9
**Knowledge score (categories)** (37 missing)	
Low/Intermediate	106 (24.3%)
High	331 (75.7%)

^§^ MSc: Master of Science; PhD: Doctor of Philosophy.

**Table 2:** Descriptive analysis and crude logistic regression regarding influenza maternal immunization.

	Vaccine Uptake During Pregnancy	Crude Logistic	Adjusted Logistic	
		**Odds Ratio (OR) [95% Confidence Intervals (CI)]**	**OR (95% CI)**	***p*** **-Value**
**Infant’s age**		1.33 (0.96–1.83)	-	-
Mean (SD) and Median (IQR) for Women with no Vaccine Uptake (*n* = 214)	0.7 (0.6) and 0.5 (0.2–0.9)	-	-	-
Mean (SD) and Median (IQR) for Women with Vaccine Uptake (*n* = 187)	0.8 (0.6) and 0.7 (0.3–1.0)	-	-	-
	*n* (%)			
**Maternal age (groups)**				
<25	5 (23.8%)	1	1	-
25–29	27 (37.0%)	1.88 (0.62–5.70)	1.55 (0.30–7.99)	0.603
30–34	74 (52.1%)	3.48 (1.21–10.02) *	2.35 (0.48–11.57)	0.292
≥35	104 (48.1%)	2.97 (1.05–8.40) *	1.24 (0.26–5.97)	0.793
**Nationality**				
Greek	193 (49.2%)	2.57 (1.48–4.46) *	1.00 (0.41–2.45)	0.998
Other	20 (27.4%)	1	1	
**Number of children**				
1	82 (45.3%)	1	-	-
2	92 (50.3%)	1.22 (0.81–1.84)	-	-
≥3	17 (34.7%)	0.64 (0.33–1.24)	-	-
**Season of labor**				
Winter	50 (48.5%)	1	1	-
Spring	65 (58.6%)	2.29 (1.03–5.07) *	2.29 (1.03–5.07)	0.042 *
Summer	49 (43.0%)	0.95 (0.45–2.03)	0.95 (0.45–2.03)	0.900
Autumn	40 (35.4%)	0.59 (0.27–1.29)	0.59 (0.27–1.29)	0.189
**Living region**				
Athens	85 (50.9%)	1	-	-
Another Greek city	75 (42.6%)	0.72 (0.47–1.10)	-	-
Another Greek town	47 (43.9%)	0.76 (0.46–1.23)	-	-
**Family state**				
Unmarried	7 (25.0%)	1	1	
Married/cohabitation agreement	204 (47.2%)	2.68 (1.12–6.45) *	0.73 (0.18–2.92)	0.660
Divorced/estranged	3 (42.9%)	2.25 (0.40–12.62)	1.17 (0.10–14.32)	0.904
**Insurance**				
No	2 (11.8%)	1	1	
Yes	212 (47.0%)	6.65 (1.50–29.43) *	1.27 (0.17–9.40)	0.814
**Are you considered a high-risk group?**				
No	183 (44.5%)	1	-	-
Yes	7 (70.0%)	2.91 (0.74–11.40)	-	-
**Maternal education level **				
School graduate	55 (38.2%)	1	1	
Technical school graduate	34 (41.5%)	1.15 (0.66–1.99)	1.33 (0.58–3.04)	0.495
University graduate	53 (42.7%)	1.21 (0.74–1.97)	0.98 (0.46–2.10)	0.958
MSc/PhD **^§^**	73 (64.6%)	2.95 (1.77–4.93) *	2.93 (1.23–7.00)	0.015 *
**Paternal education level**				
School graduate	77 (36.8%)	1	-	-
Technical school graduate	41 (50.6%)	1.76 (1.05–2.95) *	-	-
University graduate	51 (54.8%)	2.08 (1.27–3.42) *	-	-
MSc/PhD **^§^**	42 (56.8%)	2.25 (1.31–3.86) *	-	-
**Mother’s profession **				
Public worker	42 (57.5%)	1	1	
Private worker	92 (45.8%)	0.62 (0.36–1.07)	0.48 (0.21–1.06)	0.071
Freelancer	31 (44.9%)	0.60 (0.31–1.17)	0.86 (0.31–2.39)	0.775
Unemployed	24 (43.6%)	0.57 (0.28–1.16)	0.94 (0.31–2.93)	0.922
Other	25 (35.2%)	0.40 (0.20–0.79) *	0.91 (0.30–2.71)	0.864
**Father’s profession**				
Public worker	39 (58.2%)	1	-	-
Private worker	107 (46.3%)	0.62 (0.36–1.07)	-	-
Free lancer	56 (40.9%)	0.50 (0.27–0.90) *	-	-
Other	8 (32.0%)	0.34 (0.13–0.89) *	-	-
**HCP’s recommendation regarding influenza vaccination during pregnancy**				
No	13 (9.0%)	1	1	
Yes	201 (62.4%)	16.87 (9.14–31.13) *	18.86 (8.61–41.31)	<0.001 *
**Categories Knowledge score**				
Low/Intermediate	11 (10.4%)	1	-	-
High	192 (58.0%)	11.93 (6.16–23.11) #	-	-
**Are you afraid of possible vaccines’ adverse events during pregnancy**				
No/a little afraid	104 (58.8%)	2.39 (1.63–3.50) *	2.09 (1.18–3.70)	0.012 *
Fairly afraid/very afraid	108 (37.4%)	1	1	
**Considering influenza disease dangerous for pregnant women **				
**Non-dangerous/relatively non-dangerous**	30 (22.2%)	1	1	
Relatively dangerous/Very dangerous	185 (55.4%)	4.35 (2.74–6.88) *	8.05 (3.81–17.03)	<0.001 *
**Considering influenza disease dangerous for infants**				
**Non-dangerous/relatively non-dangerous**	9 (18.0%)	1	1	
Relatively dangerous/Very dangerous	206 (49.2%)	4.41 (2.09–9.29) *	0.89 (0.29–2.72)	0.838
**Considering influenza vaccine dangerous for pregnant women**				
**Non-dangerous/relatively non-dangerous**	185 (55.9%)	4.43 (2.80–7.03) *	4.37 (2.27–8.41)	<0.001 *
Relatively dangerous/Very dangerous	30 (22.2%)	1	1	

* statistically different OR from that of reference category (*p* < 0.05). ^§^ MSc: Master of Science; PhD: Doctor of Philosophy. # not included in the multiple logistic model due to multicollinearity with other factors in the model (sub-questions of knowledge score).

**Table 3:** Descriptive analysis and crude logistic regression regarding pertussis maternal immunization.

	Vaccine Uptake During Pregnancy	Crude Logistic	Adjusted Logistic	
		OR (95% CI)	OR (95% CI)	*p*-Value
Infant’s age		0.85 (0.54–1.35)	-	-
Mean (SD) and Median (IQR) for Women with no Vaccine Uptake (*n* = 214)	0.7 (0.6) and 0.6 (0.3–1.0)	-	-	-
Mean (SD) and Median (IQR) for Women with Vaccine Uptake (*n* = 187)	0.7 (0.5) and 0.6 (0.3–0.9)	-	-	-
	*n* (%)			
Maternal age (groups)				
<25	4 (21.0%)	1	-	-
25–29	10 (13.9%)	0.60 (0.17–2.20)	-	-
30–34	29 (20.4%)	0.96 (0.30–3.12)	-	-
≥35	32 (14.9%)	0.66 (0.21–2.11)	-	-
**Nationality**				
Greek	68 (17.5%)	1.31 (0.64–2.69)	-	-
Other	10 (13.9%)	1	-	-
**Number of children**				
1	38 (21.2%)	1	1	
2	23 (12.7%)	0.54 (0.31–0.95) *	0.55 (0.27–1.14)	0.111
≥3	6 (12.2%)	0.52 (0.21–1.31)	0.52 (0.17–1.62)	0.261
**Season of labor**				
Winter	19 (18.6%)	1.20 (0.59–2.43)	-	-
Spring	19 (17.3%)	1.09 (0.54–2.21)	-	-
Summer	17 (14.9%)	0.92 (0.45–1.88)	-	-
Autumn	18 (16.1%)	1	-	-
**Living region**				
Athens	29 (17.6%)	1	-	-
Another Greek city	35 (20.1%)	1.18 (0.68–2.04)	-	-
Another Greek town	12 (11.3%)	0.60 (0.29–1.23)	-	-
**Family state**				
Unmarried	3 (10.7%)	-	-	-
Married/cohabitation agreement	75 (17.6%)	-	-	-
Divorced/estranged	0 (0.0%)	-	-	-
**Insurance**				
No	0 (0.0%)	-	-	-
Yes	78 (17.4%)	-	-	-
**Are you considered a high-risk group;**				
No	66 (16.2%)	1	-	-
Yes	1 (10.0%)	0.57 (0.07–4.61)	-	-
**Maternal education level**				
School graduate	19 (13.4%)	1	-	-
Technical school graduate	19 (23.5%)	1.98 (0.98–4.02)	-	-
University graduate	19 (15.3%)	1.17 (0.59–2.33)	-	-
MSc/PhD **^§^**	21 (18.7%)	1.49 (0.76–2.94)	-	-
**Paternal education level**				
School graduate	32 (15.4%)	1	**-**	**-**
Technical school graduate	16 (20.0%)	1.38 (0.71–2.67)	**-**	**-**
University graduate	17 (18.5%)	1.25 (0.65–2.38)	**-**	**-**
MSc/PhD **^§^**	12 (16.4%)	1.08 (0.52–2.23)	**-**	**-**
**Mother’s profession **				
Public worker	9 (12.7%)	1	**-**	**-**
Private worker	38 (19.0%)	1.62 (0.74–3.54)	**-**	**-**
Freelancer	10 (14.5%)	1.17 (0.44–3.08)	**-**	**-**
Unemployed	10 (18.9%)	1.60 (0.60–4.27)	**-**	**-**
Other	11 (15.5%)	1.26 (0.49–3.26)	**-**	**-**
**Father’s profession **				
Public worker	13 (19.4%)	1	-	-
Private worker	39 (17.2%)	0.86 (0.43–1.73)	-	-
Free lancer	21 (15.3%)	0.75 (0.35–1.61)	-	-
Unemployed	4 (16.7%)	0.83 (0.24–2.85)	-	-
Other	13 (19.4%)	1	-	-
**HCP’s recommendation regarding pertussis vaccination during pregnancy**				
No	14 (4.2%)	1	1	
Yes	64 (52.0%)	25.03 (13.18–47.53)	29.55 (14.11–61.92)	<0.001 *
**Categories Knowledge score**				
Low/Intermediate	4 (3.8%)	1		
High	71 (21.6%)	6.95 (2.47–19.52) #		
**Are you afraid of possible vaccines’ adverse events during pregnancy**				
No/a little afraid	38 (21.6%)	1.74 (1.06–2.84) *	1.85 (0.93–3.67)	0.081
Fairly afraid/very afraid	39 (13.7%)	1	1	
**Considering pertussis disease dangerous for pregnant women **				
**Non-dangerous/relatively non-dangerous**	5 (5.1%)	1	1	
Relatively dangerous/Very dangerous	73 (20.2%)	4.71 (1.85–12.02) *	6.00 (1.89–19.05)	0.002 *
**Considering pertussis disease dangerous for infants**				
**Non-dangerous/relatively non-dangerous**	3 (7.3%)	1	-	-
Relatively dangerous/Very dangerous	75 (17.9%)	2.77 (0.83–9.21)	-	-
**Considering pertussis vaccine dangerous for pregnant women**				
**Non-dangerous/relatively non-dangerous**	61 (19.1%)	1.72 (0.97–3.08)	-	-
Relatively dangerous/Very dangerous	17 (12.1%)	1	-	-

* statistically different OR from that of reference category (*p* < 0.05). ^§^ MSc: Master of Science; PhD: Doctor of Philosophy. # not included in the multiple logistic model due to multicollinearity with other factors in the model (sub-questions of knowledge score).

## Data Availability

Data supporting the reported results are available as anonymized databases upon request.

## Supplementary Materials

The following supporting information can be downloaded at: https://www.mdpi.com/article/10.3390/vaccines13040347/s1, Table S1: Crude and adjusted logistic regression regarding factors influencing antenatal immunization against both influenza and pertussis.

## Abbreviations

The following abbreviations are used in this manuscript:

WHO	World Health Organization
CDC	Centers for Disease Control and Prevention
Tdap	tetanus, diphtheria, and acellular pertussis vaccine
KAP study	Knowledge–Attitudes–Practices study
CLEO	Collaborative Center for Clinical Epidemiology and Outcomes Research
SD	Standard Deviation
OR	Odds Ratio
CI	Confidence Intervals
MSc	Master of Science
PhD	Doctor of Philosophy
HCP	Healthcare practitioner/provider/professional
IQR	Interquartile Range
