# Supplementary material for: Knowledge, Attitudes, and Practices Regarding Influenza and Pertussis Immunization During Pregnancy in Greece
Source: Vaccines (Basel). 2025 Mar 25;13(4):347. doi: 10.3390/vaccines13040347 (PMC12030942; doi:10.3390/vaccines13040347)
Supplement: Supplementary file 1 [file vaccines-13-00347-s001.zip › vaccines-3479823-supplementary.pdf]

Supplementary Material:
Table S1: Crude and Adjusted logistic regression regarding factors influencing antenatal immunization against both influenza and pertussis

	Yes (N=62)	Crude Logistic		Adjusted Logistic	
	n (%)	OR (95% CI)	p-value	OR (95% CI)	p-value
**Maternal age**	N=60				
Mean (sd)	33.8 (4.4)			-	-
Median (IQR)	34 (31-37)	1.00 (0.95-1.05)	0.957	-	-
**Infant's age**	N=48				
Mean (sd)	0.7 (0.4)			-	-
Median (IQR)	0.7 (0.3-0.9)	0.86 (0.51-1.43)	0.558	-	-
**Maternal age (groups)**					
<25	2 (10.0%)	1		-	-
25-29	9 (12.3%)	1.27 (0.25-6.39)	0.776	-	-
30-34	24 (16.9%)	1.83 (0.40-8.41)	0.437	-	-
>=35	25 (11.7%)	1.19 (0.26-5.44)	0.822	-	-
**Nationality**					
Greek	54 (13.9%)	1.31 (0.59-2.87)	0.507	-	-
Other	8 (11.0%)	1		-	-
**Number of children**					
1	28 (15.6%)	1		-	-
2	21 (11.6%)	0.71 (0.39-1.31)	0.274	-	-
>=3	4 (8.2%)	0.48 (0.16-1.45)	0.194	-	-
**Season of labor**					
Winter	18 (17.6%)	1.99 (0.89-4.44)	0.094	-	-
Spring	16 (14.5%)	1.58 (0.70-3.57)	0.274	-	-
Summer	13 (11.4%)	1.19 (0.51-2.79)	0.683	-	-
Autumn	11 (9.7%)	1		-	-

**Living region**					
Athens	23 (13.9%)	1		-	-
Another Greek city	26 (14.9%)	1.09 (0.60-2.00)	0.775	-	-
Another Greek town	11 (10.3%)	0.71 (0.33-1.53)	0.384	-	-
**Family state**					
Unmarried	3 (10.7%)	-	-	-	-
Married/cohabitation agreement	59 (13.8%)	-	-	-	-
Divorced/Estranged	0 (0.0%)	-	-	-	-
**Insurance**					
No	0 (0.0%)	-	-	-	-
Yes	62 (13.8%)	-	-	-	-
**Are you considered a high-risk group;**					
No	51 (12.5%)	1		-	-
Yes	1 (10.0%)	0.78 (0.10-6.27)	0.813	-	-
**Maternal education level**					
School graduate	13 (9.1%)	1		1	
Technical school graduate	15 (18.5%)	2.27 (1.02-5.06)	**0.044***	2.30 (0.85-6.25)	0.101
University graduate	15 (12.1%)	1.38 (0.63-3.02)	0.425	1.35 (0.51-3.56)	0.547
MSc/PhD§	19 (17.0%)	2.04 (0.96-4.34)	0.063	2.12 (0.82-5.43)	0.12
**Paternal education level**					
School graduate	21 (10.1%)	1		-	-
Technical school graduate	15 (18.5%)	2.02 (0.99-4.16)	0.055	-	-
University graduate	15 (16.3%)	1.73 (0.85-3.54)	0.13	-	-
MSc/PhD§	10 (13.7%)	1.41 (0.63-3.16)	0.4	-	-
**Mother's profession**					
Public worker	9 (12.5%)	1		-	-

Private worker	29 (14.5%)	1.19 (0.53-2.65)	0.675	-	-
Free lancer	8 (11.6%)	0.92 (0.33-2.53)	0.869	-	-
Unemployed	10 (18.5%)	1.59 (0.60-4.24)	0.353	-	-
Other	6 (8.5%)	0.65 (0.22-1.92)	0.432	-	-
**Father's profession**					
Public worker	11 (16.4%)	1		-	-
Private worker	32 (14.0%)	0.83 (0.39-1.74)	0.618	-	-
Free lancer	17 (12.4%)	0.72 (0.32-1.64)	0.436	-	-
Unemployed	1 (4.2%)	0.22 (0.03-1.81)	0.16	-	-
**HCP's recommendation regarding influenza vaccination during pregnancy**					
No	6 (4.1%)	1		1	
Yes	56 (17.6%)	4.93 (2.07-11.73)	**<0.001***	1.09 (0.36-3.26)	0.878
**HCP's recommendation regarding pertussis vaccination during pregnancy**					
No	12 (3.5%)	1		1	
Yes	50 (40.7%)	18.66 (9.46-36.81)	**<0.001***	19.97 (9.08-43.94)	**<0.001***
**Knowledge score**	N=60				
Mean (sd)	8.4 (1.0)	1.70 (1.32-2.18)	**<0.001***	-	-
Median (IQR)	9 (7.5-9)			-	-
**Categories Knowledge score**					
Low/Intermediate	3 (2.9%)	1		-	-
High	57 (17.3%)	7.10 (2.17-23.17)	**0.001***	-	-
**Are you afraid of possible vaccines' adverse events during pregnancy**					
No/a little afraid	34 (19.2%)	2.28 (1.32-3.93)	**0.003***	2.45 (1.20-5.01)	**0.014***

Fairly afraid/very afraid	27 (9.4%)	1		1	
**Considering influenza disease dangerous for pregnant women**					
Non-dangerous/relatively non-dangerous	2 (1.5%)	1		1	
Relatively dangerous/ Very dangerous	60 (18.1%)	14.56 (3.50-60.48)	**<0.001***	11.99 (2.39-60.08)	**0.003***
**Considering pertussis disease dangerous for pregnant women**					
Non-dangerous/relatively non-dangerous	4 (4.0%)	1		1	
Relatively dangerous/ Very dangerous	58 (16.1%)	4.55 (1.61-12.85)	**0.004***	1.85 (0.52-6.55)	0.343
**Considering influenza disease dangerous for infants**					
Non-dangerous/relatively non-dangerous	0 (0.0%)	-	-	-	-
Relatively dangerous/ Very dangerous	62 (14.9%)	-	-	-	-
**Considering pertussis disease dangerous for infants**					
Non-dangerous/relatively non-dangerous	3 (7.3%)	1		-	-
Relatively dangerous/ Very dangerous	59 (14.1%)	2.08 (0.62-6.94)	0.236	-	-
**Considering influenza vaccine dangerous for pregnant women**					
Non-dangerous/relatively non-dangerous	50 (15.2%)	1.84 (0.95-3.58)	0.071	-	-
Relatively dangerous/ Very dangerous	12 (8.9%)	1		-	-

**Considering pertussis vaccine dangerous for pregnant women**					
Non-dangerous/relatively non-dangerous	50 (15.6%)	1.99 (1.02-3.87)	**0.042***	1.36 (0.59-3.18)	0.472
Relatively dangerous/ Very dangerous	12 (8.5%)	1		1	
**When did the questionnaire was completed?**					
Before COVID-19 vaccine implementation	45 (14.2%)	1		-	-
After COVID-19 vaccine implementation	14 (11.2%)	0.77 (0.40-1.45)	0.412	-	-

*statistically different OR from that of reference category (p<0.05)

§MSc: Master of Science; PhD: Doctor of Philosophy

#not included in the multiple logistic model due to multicollinearity with other factors in the model (sub-questions of knowledge score)
